# Linking rapid erosion of the Mekong River delta to human activities

**DOI:** 10.1038/srep14745

**Published:** 2015-10-08

**Authors:** Edward J. Anthony, Guillaume Brunier, Manon Besset, Marc Goichot, Philippe Dussouillez, Van Lap Nguyen

**Affiliations:** 1 Aix-Marseille Univ., CEREGE UMR 34, 13545 Aix en Provence, France, Institut Universitaire de France; 2 Lead Sustainable Hydropower & River Basin Management, WWF Greater Mekong, Ho Chi Minh City, Vietnam; 3 HCMC Institute of Resources Geography, Vietnam Academy of Science and Technology (VAST), 01, Mac Dinh Chi Str., Dist. 01, HoChiMinh City, Vietnam

## Abstract

As international concern for the survival of deltas grows, the Mekong River delta, the world’s third largest delta, densely populated, considered as Southeast Asia’s most important food basket, and rich in biodiversity at the world scale, is also increasingly affected by human activities and exposed to subsidence and coastal erosion. Several dams have been constructed upstream of the delta and many more are now planned. We quantify from high-resolution SPOT 5 satellite images large-scale shoreline erosion and land loss between 2003 and 2012 that now affect over 50% of the once strongly advancing >600 km-long delta shoreline. Erosion, with no identified change in the river’s discharge and in wave and wind conditions over this recent period, is consistent with: (1) a reported significant decrease in coastal surface suspended sediment from the Mekong that may be linked to dam retention of its sediment, (2) large-scale commercial sand mining in the river and delta channels, and (3) subsidence due to groundwater extraction. Shoreline erosion is already responsible for displacement of coastal populations. It is an additional hazard to the integrity of this Asian mega delta now considered particularly vulnerable to accelerated subsidence and sea-level rise, and will be exacerbated by future hydropower dams.

River deltas crucially depend on sustained sediment supplies in order to maintain delta shoreline position and to balance subsidence. Because they are increasingly starved of sediment trapped behind dam reservoirs, many of the world’s river deltas are becoming vulnerable to accelerated subsidence and erosion, losing large tracts of land and becoming more exposed to flooding and sea-level rise^1^,^2^. This growing vulnerability has significant political, economic and environmental consequences for many of the world’s deltas, and calls for strong coordinated international efforts in terms of research and policy geared towards maintaining or restoring delta sustainability^3^,^4^. These concerns are embodied, for instance, in the International Council for Science’s (ICSU) endorsement of the initiative ‘Sustainable Deltas 2015’.

Nearly a generation after other large Asian river deltas, rendered vulnerable to erosion, sea-level rise and flooding by dams constructed in the 1970 s and 1980 s^5^, the Mekong delta now faces a major sustainability challenge. The Mekong River basin (Fig. 1) is 12^th^ in size in world rankings and drains six countries. It also has the world’s third largest delta^6^. The Mekong delta hosts a population of nearly 20 million^7^. Crucial to the food security of Southeast Asia, it provides 50% of Vietnam’s food^8^. Significantly, it accounts for 90% of Vietnam’s rice production making this country the world’s second most important rice exporter, and 60% of its seafood, both with export values of several billion US$. Furthermore, the delta is a very active area for overall agriculture and animal husbandry^8^. The delta is also the terminus of a river that has the most concentrated fish biodiversity per unit area of any large river basin in the world. It ranks second only to the Amazon in overall biodiversity^9^.

These important advantages are increasingly threatened by a number of rapid drivers of development, notably planned large-capacity dams^10^ (Fig. 1b) that are rendering the Mekong delta an iconic example of an economic, social, political and environmental hotspot. The extent to which hydropower dams are expected to affect the lower Mekong basin countries has come to the fore, especially after commencement, in November 2012, of the construction of the Xayaburi dam (reservoir capacity: 1.3 km^3^) in Lao PDR, amidst international concern and protest from the Government of Vietnam, and from scientists and environmental awareness groups^11^,^12^. The hydropower dam issue has been thoroughly discussed in a number of studies in terms of its potential social, political and ecological impacts^13^,^14^,^15^,^16^, and of the crucial problem of sediment-trapping and its consequences on the future geomorphic stability of the delta^17^,^18^,^19^,^20^,^21^,^22^. In addition to the problems expected from hydropower dams, large-scale aggregate mining in the beds of the mainstem Mekong River and distributary channels of its populous delta in Cambodia and Vietnam (Fig. 1b) has steadily increased since 2000, spurred by strong development pressures^23^,^24^. The pernicious effects of this activity on the environment^23^ have tended to receive much less attention than those of hydropower dams.

In the wake of this concern regarding the effects of dams, and, to a lesser extent, of river-bed mining, on fluvial sediment supply and on the future stability of the Mekong delta, erosion of the delta’s shoreline has become a particularly important issue, highlighted in recent academic studies^25^,^26^,^27^,^28^,^29^,^30^ and in numerous newspaper reports^31^. It has been shown from analysis of maps and Landsat satellite images spanning the period 1950–2014 that delta erosion has progressively increased, especially along the muddy South China Sea coast, whereas the delta distributary mouths sector has shown a fluctuating trend tentatively attributed to shifts in flood discharge levels and associated sediment supply^29^. In combination with subsidence, which has been shown to have been accelerated by massive groundwater extraction in this populous delta^32^, coastal erosion exacerbates the vulnerability of the delta. It poses threats to the safety and livelihood of subsistence farmers and fishers^33^, as shown by the relocation of over 1200 households in coastal settlements affected by severe erosion in 2014^31^, and the common recourse to the Vietnamese army in setting up hasty coastal defences along eroding sectors of the delta in the South China Sea.

The vulnerability of the Mekong delta thus involves a conjunction of various hot issues that are attracting international scientific and political attention, underpinned by the tensions raised by the planned large hydropower dam projects^11^,^12^,^14^, and the threats such projects pose for the sustainability of the world’s river deltas^3^,^4^. Here, we focus on the important issue of the erosion of the Mekong delta. First, we analyse recent high-resolution satellite images spanning nearly a decade to provide a precise picture of the state-of-health of the delta’s shoreline. We then explore the direct and indirect mechanistic links between delta erosion and the impacts of some of the human activities and effects evoked above, notably a decreasing sediment supply. The scale and breadth of these activities in the Mekong basin and delta, compounded by the less clearly identified effects of climate change^7^,^34^,^35^, mediate coastal erosion of the delta in complex ways that still need to be clearly elucidated. Quantifying the scale and rates of coastal erosion, and identifying how such erosion is mechanistically linked to human activities, are important steps in assessing the increasing vulnerability of this mega delta, and in the search for solutions aimed at mitigating such vulnerability.

## Late Holocene growth and physiography of the Mekong River delta

The Mekong delta prograded rapidly in a relatively sheltered bight in the South China Sea under the influence of high fluvial sediment supply 5300 to 3500 years ago, developing from an estuary into a delta^36^,^37^. This >200 km seaward growth resulted in increasing exposure of the delta to ocean waves that led to a more wave-influenced mode of progradation characterised by the construction of numerous sets of beach ridges in the sector of the distributary mouths^26^. Under this increasingly wave-influenced regime, the rate of seaward delta growth over the last 3000 years has been of the order of 16 m/year in this sandy beach-ridge dominated sector of the delta, while at the same time, westward longshore transport of much of the muddy load debouching at the mouths has resulted in a progradation rate of up to 26 m/year in the Ca Mau sector (Fig. 2a) in the southwest^36^,^37^,^38^. The lower Mekong delta is thus characterised by two dominant coastal landform types, numerous sandy beach-ridge sets with large inter-ridge depressions of sand and finer sediment along a 250 km stretch of coast from the multiple distributary mouths to Bac Lieu, and a prograded mud-dominated coast westwards of Bac Lieu that forms the remaining 350 km of shoreline along the rest of the South China Sea and in the Gulf of Thailand (Fig. 2a).

**Figure 1:**
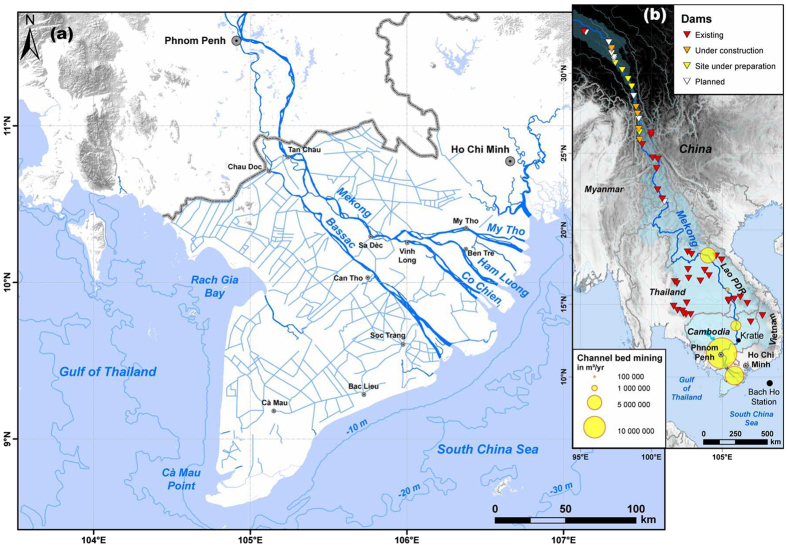
The Mekong River delta in Vietnam, the world’s third largest delta. (**a**) The delta covers an area of about 60,000 km^2^ and comprises a dense network of canals and dykes, some of which are shown here. The map was drawn from base maps of National Geographic and Esri (Source: http://goto.arcgisonline.com/maps/NatGeo_World_Map). Map projection is in UTM 48 N coordinates with WGS 84 datum. The hydrographic network and bathymetry were drawn from^59^. Canals were drawn from NatGeo_World_Map in ESRI ArcGIS 10.2 Desktop. (**b**) Map with relief, derived from^59^, shows five of the six Mekong river basin countries and existing and planned dams. Country boundaries were drawn from the *World Countries* dataset showing the boundaries as they existed in December 2013 (Source: Esri, DeLorme Publishing Company, CIA World Factbook). Dams were mapped from data provided by^10^,^11^,^14^,^18^.

**Figure 2:**
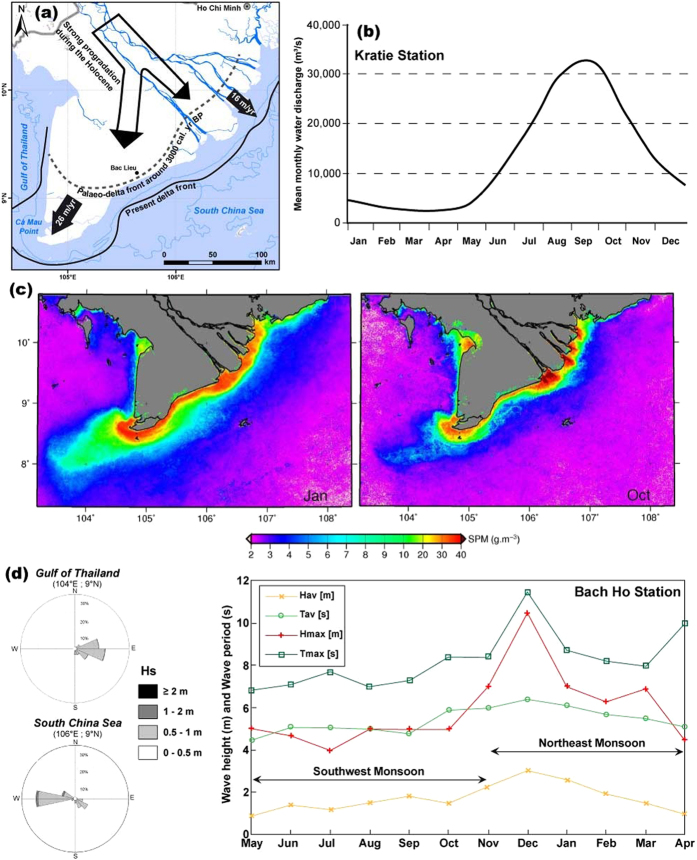
Progradation, discharge, delta-front sediment dynamics, and hydrodynamic setting of the Mekong River delta. (**a**) Gross progradation over the last 3000 years (adapted from^38^, after^37^, with permission from Elsevier; base map from National Geographic and Esri (Source: http://goto.arcgisonline.com/maps/NatGeo_World_Map); hydrographic network and bathymetry on base map were derived from^59^; (**b**) Monthly water discharge at Kratie (see Fig. 1b) from data provided by^7^; **(c**) Suspended particulate matter (SPM) in the coastal zone off the Mekong delta estimated from the MEdium Resolution Imaging Spectrometer (*MERIS*) on board the Envisat satellite platform^46^ (with permission from Elsevier). The SPM concentrations were obtained from about 2000 MERIS images covering the period 2003–2012, which coincides with the years covered by the SPOT satellite imagery used to monitor shoreline change. The authors used the MERIS third reprocessing as the input parameter in various algorithms that have been validated against extensive *in situ* datasets collected in various coastal waters and off the Mekong delta in March 2012 to convert the remote sensing reflectance, *R*_rs_ into SPM or b_bp_. The spatio-temporal patterns of SPM and b_bp_ retrieved by the authors from these different algorithms are highly coherent due to the fact that b_bp_ variability in coastal waters is driven by the SPM concentration variability to a first order. Monotonic changes of SPM and b_bp_ over the period investigated by the authors were assessed from non‐parametric seasonal Kendall statistics on the SPM and b_bp_ monthly temporal series. This test is robust against non‐normality, missing data and extreme values, and accounts for the presence of seasonality in the series. The images show a strong seasonal climatology of concentrations in October (high river-discharge season, supply to the sea) and January (low river discharge, coastal transport westward); (**d**) Wave roses for the Gulf of Thailand and South China Sea (Wavewatch III data from National Center for Environmental Prediction (NCEP): http://polar.ncep.noaa.gov/waves/download.shtml?) and monthly wave parameters (average (av) and maximum (max) wave heights (H) and periods (T)) from the Bach Ho Island Station (see Fig. 1b) located 150 km offshore of the mouths of the Mekong (data from^30^ with permission from the Coastal Education and Research Foundation).

The mean water discharge of the Mekong at Kratie, in Cambodia (Fig. 1b) is 14,500 m^3^/s^7^. The annual hydrological regime is seasonal (Fig. 2b) with a southwest Monsoon flood season (May-October) during which river-borne sediment is delivered to the delta and coastal ocean through several distributary mouths associated with the two main branches, the Bassac and the Mekong (Fig. 1a). Estimates of the mean annual suspended sediment load of the Mekong are uncertain. Depending on limited measurements and on the methods of computation, these estimates range from 50 to 160 Mt^17^,^18^,^19^,^39^,^40^,^41^,^42^. This large range variability is also reflected in the uncertainty regarding the amount of sediment trapped behind existing dams, which has been quantified as ranging from relatively significant^18^ to negligible^19^. The bedload in transit at Kratie has been estimated at about 3 Mt a year^41^. The amount of sediment deposited in the Mekong delta plain in Vietnam has been estimated as ranging from 1% in a low flood year to 6% in a high flood year relative to the total sediment load at Kratie^21^. Similar estimates for the Cambodian part of the delta range from 19 to 23%. During the high-flow southwest Monsoon season, the fraction of mud transported to the sea has been estimated as ranging from 48 to 60% of the total load at Kratie^21^. This load is essentially stored in the nearshore area close to the distributary mouths during the high-flow season^43^,^44^,^45^,^46^, as illustrated by a 10-year mean (2003–2012) for the month of October, of suspended particulate matter (SPM) concentrations (Fig. 2c) derived from *MERIS* satellite data^46^. The shorter low-flow dry season is characterised by southwestward alongshore redistribution of part of this load, as highlighted by the 10-year *MERIS* SPM mean for January (Fig. 2c).

The Mekong delta is exposed to low-to-moderate energy waves from the southwest during the southwest Monsoon season (Fig. 2d) that generate weak longshore currents towards the northeast, a situation that favours the mud storage in the river mouth sector. The northeast Monsoon season is characterised by higher waves (Fig. 2d) responsible for the active alongshore sediment transport westwards from the mouths (Fig. 2c). This wave-induced transport is reinforced by wind stress and by tidal currents associated with a tidal range that decreases from about 3.5 m at mean spring tides along the mouths of the Mekong, where tides are semi-diurnal, to less than 1 m in the Gulf of Thailand where they are diurnal. The gulf coast of the Mekong is also relatively sheltered from the higher-energy northeast Monsoon waves. The strong westward drift of mud and the resulting massive accumulation in the lower-energy sector of the Gulf of Thailand over the last 3000 years (Fig. 2a) mediated the asymmetric shape of the delta.

## Mekong delta shoreline changes

**Figure 3:**
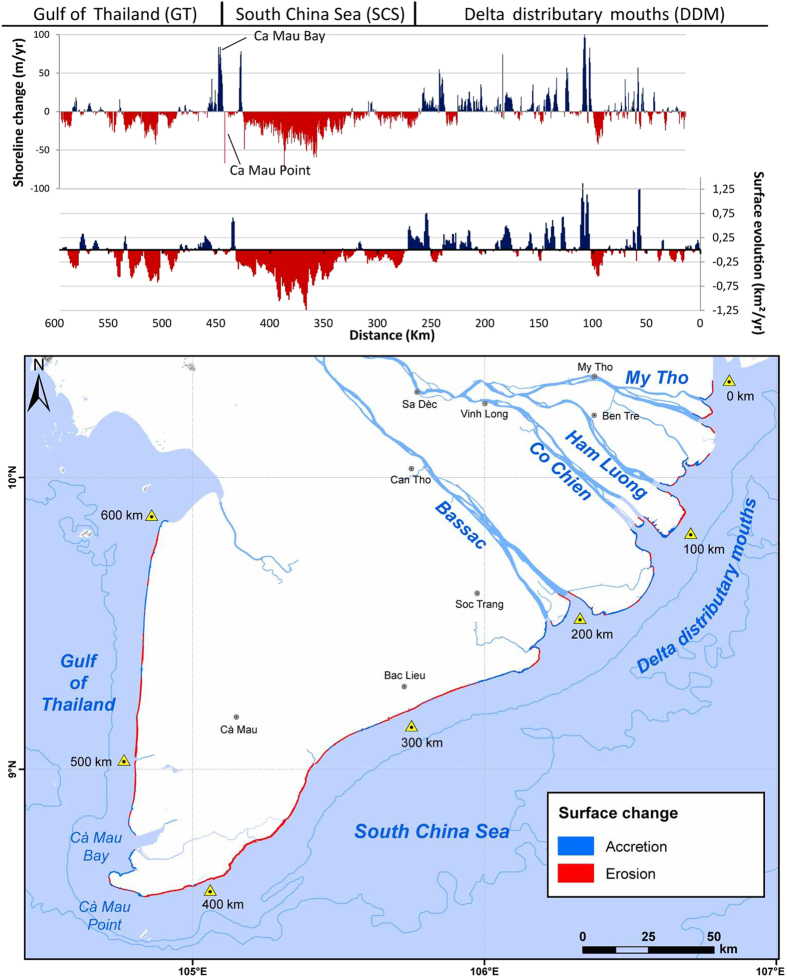
Graphs of shoreline (m/year, error ± 0.5 m/yr) and coastal area (km^2^/year, error ± 0.005 km^2^/yr) change rates for the Mekong River delta between 2003 and 2011/12 analysed from high-resolution SPOT 5 satellite images (top). The map (bottom) shows shoreline accretion and erosion sectors divided into three sectors: the sand-bound delta distributary mouth (DDM) sector comprising beaches with mildly developed aeolian dunes, the muddy South China Sea (SCS) where past deltaic progradation rates were highest, and the muddy Gulf of Thailand (GT), both colonised by mangroves increasingly replaced by shrimp farms. Erosion rates along the SCS coast increase towards the southwest with distance from the river mouths but probably also as a function of a close-to shore-normal exposure to northeast Monsoon waves in conjunction with a decreasing tidal range for the most critically eroding southwestern part. Base map from National Geographic and Esri (Source: http://goto.arcgisonline.com/maps/NatGeo_World_Map); hydrographic network and bathymetry from^59^.

**Figure 4:**
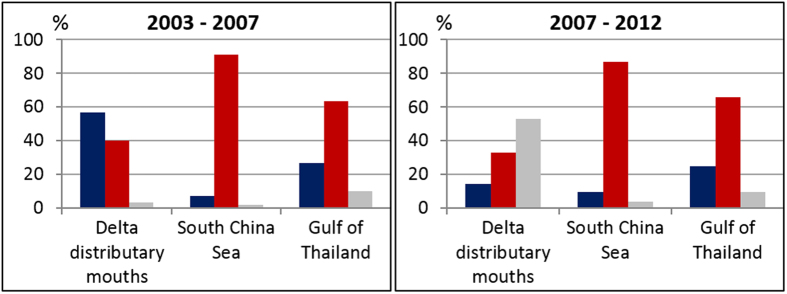
Net recent shoreline changes along the Mekong delta expressed in percentages of advance (dark blue), retreat (red) and stability (which includes the error band, grey) for the three sectors of delta coast.

The shoreline change patterns of the Mekong delta over the period 2003–2012 are described in terms of three sectors: the sand-dominated delta distributary mouths (DDM), the mud-dominated South China Sea (SCS) coast, and the mud-dominated Gulf of Thailand (GT) coast. Erosion is essentially affecting the muddy sectors with shoreline retreat rates commonly exceeding 50 m/yr in places, especially along the 180 km-long SCS coast nearly 90% of which is in retreat (Fig. 3). Over 50% of the >600 km-long Mekong delta coast has been in erosion between 2003 and 2012 but with noteworthy variations (Fig. 4). Although erosion has been less severe along the lower-energy GT coast it nevertheless concerned over 60% of this 200 km-long coast. These changes have entailed significant levels of deltaic land loss along the muddy SCS and GT coasts (Table 1) that are raising concerns in Vietnam. The delta lost over 5 km^2^ of coastal lands between 2003 and 2012, which is significant for a hitherto strongly advancing delta. The Mekong delta lost the equivalent of 1 and a half football fields every day between 2007 and 2012. This rampant erosion contrasts with the massive growth of the delta towards the southwest over the last three millennia (Fig. 2a). The net loss rate is mitigated by the sandy DDM sector, which shows mild net accretion, notwithstanding an irregular alongshore pattern of erosion and advance (Fig. 3).

The results also show interesting aspects when the two periods (2003–2007, 2006/7–2011/12) of image analysis are compared (Fig. 4, Table 1): (i) a strong decrease in accretion in the DDM sector (from 0.78 km^2^/yr to 0.26 km^2^/yr), and (ii) exacerbation of shoreline retreat and land loss along the muddy SCS sector (mean retreat rate from about 6.4 m/yr to over 12.5 m/yr throughout the 180 km of the muddy SCS sector, and land loss from 2 km^2^/yr to over 2.7 km^2^/yr). Although the net land loss decreased in the GT sector (from about 0.87 km^2^/yr to just over 0.57 km^2^/yr), erosion affected more of the coast (from 62 to 64%).

## Discussion

The high-resolution satellite images show that the hitherto strongly prograding Mekong delta is now dominated by rampant erosion. The 2003–2012 land loss rate of nearly 2.3 km^2^/year along the SCS coast (Table 1) largely exceeds a loss rate of 1.2 km^2^/year over the period 1885–1985 determined from maps^47^. The recent period has also been characterised by a swing from secular progradation of the GT coast^47^ to the present generalised erosion. The percentage of eroding delta shoreline over the period 2003–2012 has also increased from 40% between 1973 and 2003^29^ to over 50%.

**Figure 5:**
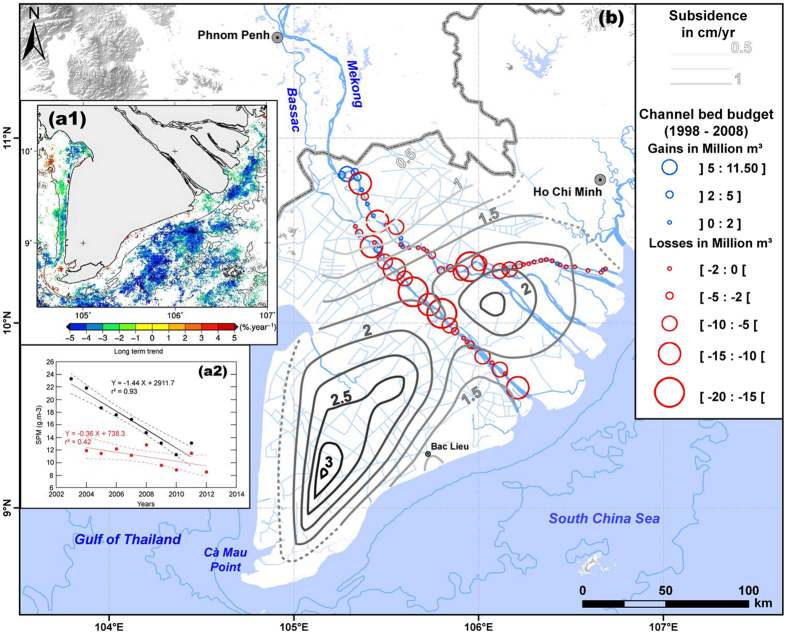
Aspects of the recent sediment balance and subsidence in the delta mediated by human activities. (**a**) Map and graph of significant monotonic trend in % per year (seasonal Kendal test, pb 0.05) of SPM off the Mekong delta^46^. Non-significant areas are shown in white. The graph shows time series of averaged SPM values as a function of year during low (red dots) and high (black dots) river flow conditions. The linear regression equations are shown for each sub-data set, with dashed lines representing the 95% confidence interval (with permission from Elsevier). The data show a net reduction of up to 5% a year in SPM off the mouths of the delta and along much of the nearshore area in the SCS attributed to dam trapping of sediment^46^. A net annual decrease in SPM of 2 to 4% is also depicted along the GT coast. (**b**) Map of the Mekong delta showing: (i) compaction-based subsidence rates redrawn from^32^. These rates are highest in the most critically eroding southwestern part of the delta; (ii) 10-year (1998–2008) bedload budget changes in the My Tho and Bassac channels, characterised by net cumulative losses of 200 Mm^3^ that have been attributed to large-scale commercial river-bed mining^48^ (with permission from Elsevier). Base map from National Geographic and Esri (Source: http://goto.arcgisonline.com/maps/NatGeo_World_Map); hydrography, relief, and bathymetry from^59^.

**Figure 6:**
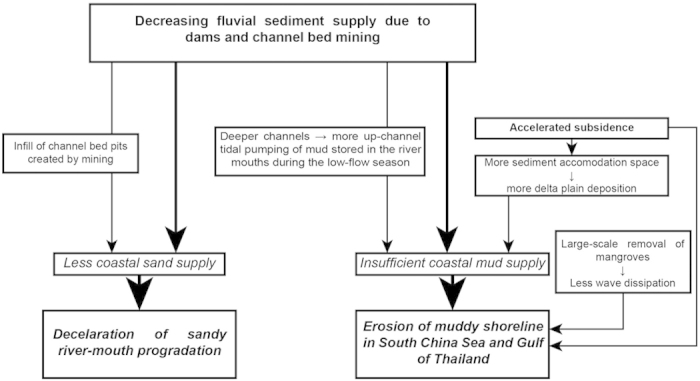
Inferred mechanistic links between coastal erosion of the Mekong delta and a human-mediated decrease in sediment available to the delta, as well as the impact of large-scale mangrove removal, to make way for shrimp farms, in particular. These links involve competition for a decreasing sediment supply marked by a sediment deficit along the coast that results in shoreline erosion. Mud and sand sequestering behind dams and large-scale riverbed sand mining are deemed to be the overarching causes in the decrease in sediment supply to the coast responsible for delta erosion. Channel mining creates pools and pits, generating deepened channels that trap sand coming from upstream in order to restore channel geometry. These extractions, and pit and pool infill, are deemed to lower the amount of sand attaining the mouths, and to be responsible for the significant slow-down in progradation of the sand-dominated mouth sector of the delta. Enhanced delta-plain deposition to fill the accommodation space created by accelerated subsidence may be having a similar effect on the balance of mud routed to the coast, potentially depriving the coastal zone of mud, and favouring accelerated muddy shoreline erosion in the GT, and especially, southern sector of the SCS. Possible seasonal but stronger tidal pumping of mud from the mud reservoir at the mouths into the artificially deepened deltaic distributary channels may also further deprive the coastal zone of mud during the high wave-energy, low-flow season.

Large deltas such as that of the Mekong are complex features the shoreline positions of which can change under the influence of a large range of factors, notably sediment supply, routing and storage, subsidence, sea level, and waves and currents. We argue here that a decreasing sediment supply is the main factor underpinning the erosion that now affects more than 300 km of the Mekong delta shoreline (Fig. 5). We also argue that ancillary mechanistic links between human-induced changes in the delta, including accelerated subsidence, and patterns of sediment routing and storage, may also be contributing to shoreline erosion (Fig. 6).

The temporal trend in SPM concentrations at the mouths of the Mekong provide a reasonable proxy highlighting a decrease in Mekong river sediment supply (Fig. 5a) in recent years^46^. Beyond the strongly seasonal variability in suspended sediments in coastal waters under the Mekong’s influence (Fig. 2c), a robustly determined long-term trend of about −5% in SPM concentration per year between 2003 and 2012 was computed from the MERIS data^46^. This annual fall in SPM was attributed to a persistent decrease in Mekong river sediment output during the critical high-flow season when the river supplies sediment to the sea^46^. For the period 1997–2012, it was further shown from analysis of significant offshore wave heights and directions (http://www.ncep.noaa.gov/), and wind speed and direction derived through cross-calibration and assimilation of ocean surface wind data from SSM/I, TMI, AMSR-E, SeaWinds on QuikSCAT, and SeaWinds on ADEOS-2 (http://podaac.jpl.nasa.gov/node/31) that this decrease in suspended sediments was not related to the hydrodynamic regime (involving, for instance, weaker sediment resuspension) in the South China Sea, which showed no significant changes over the period of analysis^46^. Furthermore, no significant changes in Mekong flood discharge likely to explain the 5% annual drop in the Mekong’s suspended sediment supply to the South China Sea between 2003 and 2012 have been found^46^,^48^.

The 2003–2012 mean MERIS coastal ocean climatology for the dry-season month of January^46^ further suggests a clear link between coastal erosion and SPM concentrations. The alongshore-uniform January pattern (Fig. 2c) represents the mud transport and resuspension belt from the river mouths and a minor (<5%) contribution by biological production^45^, but also no doubt reflects erosion^44^ of the muddy SCS shoreline (Fig. 3) under the energetic wave regime prevailing during this season (Fig. 2d). The significant role of infragravity wave energy impinging on the muddy SCS coast following gravity wave dissipation by the shoreface and mangroves has been identified^30^. This highlights the overarching role of the more energetic and longer-period northeast Monsoon waves with their larger infragravity component. Small inshore (within the 10 m isobath) zones showing an increase in the 10-year mean SPM along critically eroding areas of the SCS coast^46^ (Fig. 5a) are inconsistent with the overall 2003–2012 SPM decrease, and may, therefore, reflect sediment resuspended by chronic coastal erosion.

The recent persistent decrease in suspended sediment concentrations off the delta is attributed essentially to dam impoundment of sediment^46^, and corroborates the conclusions of a study that has quantified significant sediment retention by dams at the scale of the Mekong basin^18^. Although there is a consensus, however, on the negative impacts of existing and planned dams on the sediment supply of the Mekong to its delta^5^,^18^,^20^,^21^,^22^, the poorly estimated Mekong river load and, therefore, the uncertainty regarding what fraction of this load may be trapped behind dams, precludes linking without doubt the present delta erosion to existing dams. Dams are, not, however, the only source of a potential decrease in sediment supply to the coast. The massive channel bed mining in the Mekong (Fig. 5b), deemed to be leading to significant reductions in bedload supply to the coast^48^, should be considered a major concern in the stability of the delta’s shoreline, especially in the DDM sector, where much of the sand supplied by the river to the coast is deposited. Annual extractions were about 27 Mm^3^ (about 57 Mt) between 2008 and 2012, 86% of which was sand^24^. This rate represents nearly 20 times the annual Mekong sand flux estimated at Kratie^41^. A 10-year (1998–2008) comparison of bed depths in two of the distributary channels in the delta, the Bassac and My Tho, showed net cumulative losses of 200 Mm^3^ of bedload^48^. These losses occurred along much of the reaches of the two channels (Fig. 5b), and have been attributed to these massive channel bed sand extractions^48^. This mining activity has generated numerous pools and pits up to 15 m deeper than the natural channel bed levels in Cambodia^49^, and especially Vietnam, where the deepest pools generated between 1998 and 2008 are up to 45 m deep^48^. The numerous pits and pools created by large-scale sand mining actively trap bedload transported downstream during the high-discharge season^48^. This should be resulting in a net decrease in sand supply to the DDM sector (Fig. 6). We interpret the present irregular pattern of change in this sector (Fig. 3) as reflecting shoreline adjustments to the decreasing sand supply caused by massive mining of sand from the channel beds in the delta and upstream of the delta. This activity will increasingly impact on rates of progradation in this sector, as suggested by the decrease in shoreline advance between 2007 and 2012 (Table 1).

Another mechanism likely to be activated by sand mining is that of enhanced saltwedge intrusion in the delta channels in the dry season, a process that leads to up-channel tidal pumping of mud (Fig. 6). Up-channel transport of mud from the storage area of the mouths prevails in the lower Mekong channels during the dry season when river discharge is low and saltwater penetrates up to 40 km upstream^43^. Deeper channels favour stronger upstream intrusion of saltwater and more mud-trapping at the upstream edge of the intrusion in estuaries^50^. In the Mekong, this occurs at a time of the year when mud needs to be stored along the coast to dissipate wave energy and mitigate shoreline erosion downdrift of the DDM sector. The hypothesis of enhanced up-channel mud pumping from the coastal zone as the distributary channels in the lower Mekong delta become deeper as a result of sand mining is supported by increasing dry-season inland saltwater intrusion into the delta^47^,^51^, which has also been orally confirmed to us, especially for the lower reaches of the Bassac channel which now require almost continuous dredging of mud to maintain navigation for large vessels. The enhanced salt-wedge intrusion further poses the problem of increased salinization of cultivated land in the Mekong, especially given the accelerated subsidence caused by groundwater exploitation^32^.

Subsidence rates are highest in the southwestern sector of the delta (Fig. 5b), which essentially comprises easily compressible marshes and mud. Relatively high subsidence rates, exceeding 1.5 cm/yr, are also characteristic of the sector of coast between Bac Lieu and Ca Mau Point (Fig. 5b), which also shows the highest erosion rates in the Mekong delta (Fig. 3). Interestingly, this is also the only area of the delta where shoreline erosion has been reportedly persistent since 1885^47^. The secular erosion affecting this muddy sector largely antedates dams and the expected effects of channel-bed deepening on mud storage. This erosion may be due to a persistently weak supply of mud released from the DDM mud-storage sector as a result of delta-front sediment dynamics, but also possibly to higher incident wave energy due to a more normal shoreline orientation relative to the northeast Monsoon waves. According to a coastal sediment transport modelling study, this strongly eroding part of the delta presently receives less than 2% of the fluvial mud exiting in the sector of the mouths^44^. This finding further reinforces the argument that the January SPM along this sector of coast, shown in Fig. 2c, largely reflects coastal erosion and sediment resuspension.

Sediment partitioning and storage between the Bassac and the various Mekong distributaries to the northeast of the Bassac, more distant from this erosion hotspot, may play a role in this deficit. Aspects of mud partitioning and routing in the Mekong delta between its multiple mouths, where mud is stored during the high-season discharge before being transported alongshore, and its subaqueous front are, however, poorly known. A clearer resolution of variations in the state-of-health of the delta’s shoreline will require more comprehensive work on these aspects. If less mud is being supplied from the mouths to the rest of the delta, then it may be inferred that the lower rates of erosion of the even more distant GT coast, compared to the SCS coast (Table 1), may be due to its less energetic wave regime (Fig. 4d) and weak tidal currents associated with its low tidal range. Aspects of sand partitioning are better known. Much of the sand supplied by the river is sequestered in the DDM sector where delta progradation has been dominated by successive sets of beach ridges^26^. This trapping of sand in the DDM in the course of the formation of the Mekong delta, and up to the present, has been favoured by differential wave refraction processes generated by the highly variable shoreline morphology and bathymetry generated by a multiple river-mouth system, and by ‘hydraulic-groyne’ effects related to the water discharge from the multiple mouths^52^.

Two final but unrelated points regarding delta erosion and human activities in the populous Mekong delta are the impacts of large-scale removal of mangroves and the joint effects of accelerated subsidence and of the numerous canals on mud storage and supply to the coast (Fig. 6). The coastal mangrove system along the muddy SCS and GT coasts has been classified as ‘fringe mangrove’ occuping a narrow coastal band^30^. The vicissitudes of war and timber overexploitation have had a heavy toll on mangroves in the delta, especially heavy downcutting in the 1980 s and 1990 s to provide timber for the construction industry and charcoal, and for conversion into shrimp farms^53^,^54^. Sea dykes are also being increasingly built along parts of the muddy SCS and GT coasts for protection from marine flooding and for shrimp farms, generating a process of ‘mangrove squeeze’ and lowering of the wave-dissipating capacity of mangroves^30^. The marked alongshore variability in erosion rates of the SCS sector (Fig. 3) may reflect differences arising from the presence and protective role of mangroves or their absence which enhances erosion. However, although the dissipative role of mangroves on waves and the consequent mitigating effect on shoreline erosion along the Mekong delta have been emphasised^30^,^55^,^56^, and modelled^30^, mangrove efficiency is subordinate to sediment supply and is not sustainable under conditions of strong persistent sediment deficit, as illustrated by the mangrove-rich Guianas coast between the Amazon and Orinoco river mouths, the world’s longest muddy coast^57^. Field visits along much of the muddy SCS and GT coast during the high-energy season in 2012 confirmed active wave erosion of muddy mangrove-bearing bluffs.

Accelerated subsidence creates additional accommodation space for sediment. A supplementary effect of accelerated subsidence, therefore, besides that of contributing to exacerbated muddy shoreline erosion, may be that of potential lowering of mud supply to the sea as enhanced delta-plain deposition occurs to balance this subsidence. The numerous artificial canals in the delta plain are also likely to have an additional effect on mud supply to the coast by trapping more mud. The relationship between canals, many of which are diked, and delta-plain sedimentation is, however, far from being straightforward, especially given the large variability in such sedimentation as a function of flow volume^21^.

## Conclusion and Perspectives

High-resolution satellite images show that the Mekong delta is now largely prone to erosion, with shoreline retreat over the period 2003–2012 having affected over 50% of the >600 km-long coast, and even up to 90% of the muddy South China Sea coast. A decreasing river sediment supply to the coast is deemed to be the prime cause of this erosion, and most likely due to existing dam retention of sediment and to massive channel-bed sand mining in the delta, an activity on the increase over the last decade. An important recent decrease in mud supply to the coast during the high river-discharge season has been highlighted from MERIS staellite images^46^, whereas decreasing rates of sandy shoreline progradation in the mouths sector of the delta are in agreement with large-scale sand mining in the delta channels, including in reaches very close to the sea. Annual sand mining rates^24^ exceed by more than an order of magnitude the annual estimated bedload in transit at Kratie^41^. Sand trapping in the numerous channel bed pools and pits created by large-scale mining is expected to lower the sand supply to the beaches lining the mouths of the Mekong delta.

Subsidence accelerated by groundwater extraction is highest along parts of the muddy South China Sea coast most severely affected by erosion. The Mekong is a large complex asymmetric delta wherein competition for a decreasing sediment supply may be prevailing between the delta plain, the distributary channel beds, and the river-mouth sector where coastal mud is stored prior to redistribution towards the rest of the >600 km-long delta coast. The inferences drawn from this study suggest seasonal to persistent depletion of mud along the muddy South China Sea and Gulf of Thailand sectors of the delta’s coast. This reduction in the quantity of coastal mud results in lesser wave energy dissipation, and, consequently, in shoreline erosion. A finer clarification of the mud partitioning processes and sediment budgets involved will require, however, robust data on various aspects of sedimentation in the delta.

The uncertainty surrounding the impact of existing dams on the sediment supply to the delta is not shared by any of the future impact scenario studies. There is agreement that the planned set of future hydropower dams will definitely impact the sediment budget of the Mekong delta^18^,^20^,^21^,^22^. These dams, together with uncontrolled sand mining, will thus aggravate the on-going erosion of the delta. A recent modelling effort aimed at assessing the response of the floodplain hydrology and sediment dynamics in the delta to anthropogenic and environmental changes concluded on the overarching role of hydropower development, compared to climate change and the combined effects of sea-level rise and deltaic subsidence^21^. Operation of all the planned hydropower projects on the Mekong will reportedly increase the sediment-trapping efficiency of dam reservoirs from 11–12 Mt/year to 70–73 Mt/year^18^. Another study suggests that a cumulative sediment reduction of 51% and 96% to the delta will occur under a ‘definite future’ scenario of 38 dams (built or under construction) and full construction of all planned dams, respectively^20^. These are substantial reductions, whatever the true sediment load of the Mekong. The latter scenario implies that once sediment stored in the channels is exhausted by natural downstream transport, 96% of the pre-dam (pre-1990) sediment load would be trapped as of year 2020, by which time it is assumed that all dams are to be completed^20^. This depletion stage may be attained well before 2020 if sand mining in the delta and in the river reaches upstream is to continue at its present rate. Given the already high vulnerability of the Mekong delta, the sediment supply necessary to mitigate wave- and current-induced shoreline erosion, and balance subsidence and rising sea level, will decrease more drastically. Erosion of the sediment-starved delta coast will increase, generating further large-scale geomorphic reorganization and loss of land and resources for the world’s third largest delta.

Understanding the links between erosion of the Mekong delta and sediment supply reduction by dams, channel sand mining, subsidence, and the additional effects of competition for a decreasing sediment load between the delta plain and the shoreline, is imperative for a better apprehension of the increasing vulnerability of this mega delta. This understanding, underpinned by more reliable measurements of sediment flux, is also necessary in the search for solutions to mitigate such vulnerability.

## Methods

### Shoreline change rate

We chose available high-resolution satellite images that offered not only large individual coverage, given the length of the delta shoreline (>600 km), thus minimizing errors likely to arise from smaller areal coverage and multiple operator manipulations, but also robust and accurate determinations of shoreline change rates. A total of 43 SPOT 5, level 3, orthorectified colour satellite images available for 2003, 2006/2007 and 2011/12 at a scale of 1:10,000 were available. Although SPOT 5 images also exist for 2014 and 2015, the coverage is incomplete and we therefore chose to limit our study to the complete 2003–2012 sets. The images have a high Super-Mode 2.5 m pixel resolution obtained from two 5 m pixel resolution panchromatic images (0.48–0.71 μm) acquired simultaneously with half pixel lapse. We used the ArcMap extension module Digital Shoreline Analysis System (DSAS), version 4.3^58^, coupled with ArcGIS®10, to digitise rates of change in shoreline position. The brush/plantation fringe in sectors of sandy shoreline characterized by beaches and the mangrove fringe in the muddy sectors were adopted as good ‘shoreline’ markers, which we verified from extensive field reconnaissance in 2011 and 2012 covering over 300 km of delta shoreline. We calculated every 100 m alongshore the shore-normal distance of the vegetation line to a base line for the three sets of dates. This distance, chosen as a compromise between quality of the interpretation and total length of analysed shoreline (606 km) was then divided by the time in years between two dates to generate a shoreline change rate, the End Point Rate in DSAS 4.3. A total of 6060 change rates, each corresponding to a DSAS transect, were determined for each set of dates. We retained a relatively large uncertainty shoreline change band of ±20 m, which is much more than commonly used in the literature. We then defined the annual error (E) of shoreline change rate from the following equation:


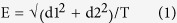


where d1 and d2 are the uncertainty estimates for the successive sets of images and T time in years between image sets. The obtained error band of ±3.5 m/yr between 2003 and 2012 was further augmented to ±5 m/yr, which we consider as an extremely cautious error range.

### Area change rate

Coastal area variations (km^2^) giving land losses or gains associated with changes in shoreline position were calculated from 1 km-alongshore segments between two successive image dates by dividing area variation by the time in years between dates. The error (ShaE/ km^2^) was calculated using a method similar to that of shoreline change rate for each 1 km segment based on the following equation:


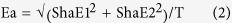


where ShaE1 and ShaE2 are the shoreline area error estimates for the successive sets of images and T time in years between image sets. The obtained area error band of ±0.0035 km^2^/yr between 2003 and 2012 was augmented to ±0.005 km^2^/yr. Shoreline and area change rates are reported on a base map derived from National Geographic and Esri (Source: http://goto.arcgisonline.com/maps/NatGeo_World_Map). The hydrography, relief and bathymetry on all maps are derived from^59^.

## Additional Information

**How to cite this article**: Anthony, E. J. *et al.* Linking rapid erosion of the Mekong River delta to human activities. *Sci. Rep.* **5**, 14745; doi: 10.1038/srep14745 (2015).

## Figures and Tables

**Table 1:** Mean yearly change rates of the Mekong delta shoreline by sector.

Sector	2003–2007		2007–2012	
	Mean cross-shore shoreline change (m/yr)	Net surface area change (km^2^/yr)	Mean cross-shore shoreline change (m/yr)	Net surface area change (km^2^/yr)
Delta distributary mouths (220 km)	+4.24	+0.78	+5.17	+0.263
South China Sea (180 km)	−6.41	−2.019	−12.53	−2.715
Gulf of Thailand (200 km)	−2.15	−0.87	−2.20	−0.575

Sector shoreline lengths are shown in parentheses.
